# Supplementary material for: Expansion of human centromeric arrays in cells undergoing break-induced replication
Source: Cell Rep. Author manuscript; Available in PMC 2024 Apr 22. (PMC11034957; doi:10.1016/j.celrep.2024.113851)
Supplement: 1 [file NIHMS1980913-supplement-1.pdf]

Cell Reports, Volume *43*

Supplemental information

Expansion of human centromeric arrays in cells undergoing break-induced replication

Soyeon Showman, Paul B. Talbert, Yiling Xu, Richard O. Adeyemi, and Steven Henikoff

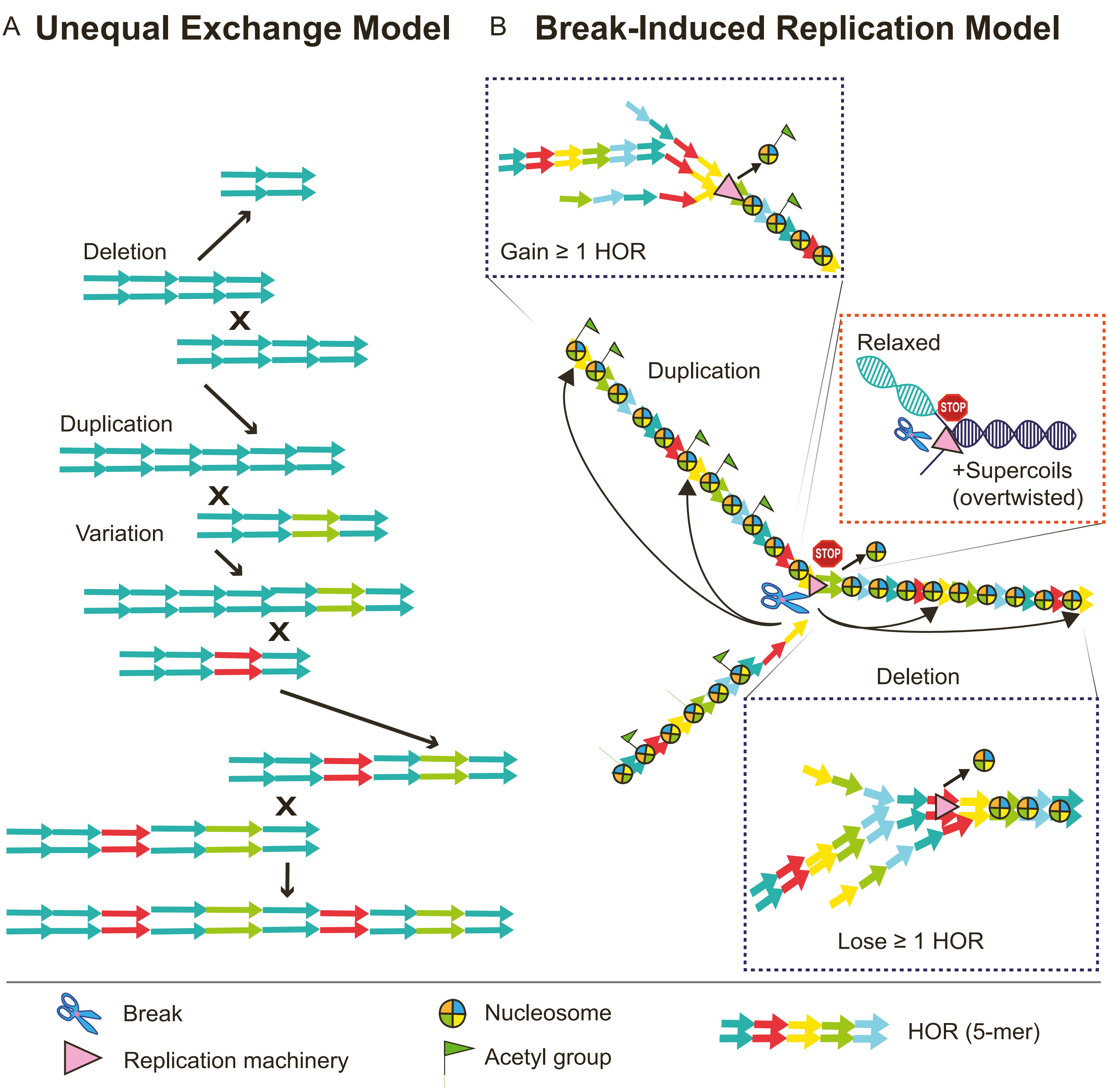
A Unequal Exchange Model
B Break-Induced Replication Model
Deletion
X
Duplication
X
Variation
X
X
Gain ≥ 1 HOR
Relaxed
STOP
+Supercoils
(overtwisted)
Duplication
STOP
Deletion
Lose ≥ 1 HOR
Break
Replication machinery
Nucleosome
Acetyl group
HOR (5-mer)

Figure S1. Schematic of two possible models that can lead to higher-order-repeats (HOR) copy number variation, relating to Introduction.

(A) In the unequal exchange model, tandem repeats such as alpha satellites can change their copy numbers by unequal exchange recombination between out-of-register paired sister chromatids resulting in either deletion or duplication of monomers. HORs can be generated when out-of-register unequal exchange occurs repeatedly between monomers that have variations. (B) In the BIR model, HORs can lead to either duplication or deletion of copies depending on the location of out-of-register re-initiation of replication with respect to the collapsed replication fork during the one-ended double strand break repair (blue dotted boxes). Duplication might occur more frequently than deletion because the chromatin behind the fork is more accessible to strand invasion owing to the new acetylated histones and/or the relaxed torsional state, in contrast to the overtwisted DNA ahead of the fork (orange dotted box).

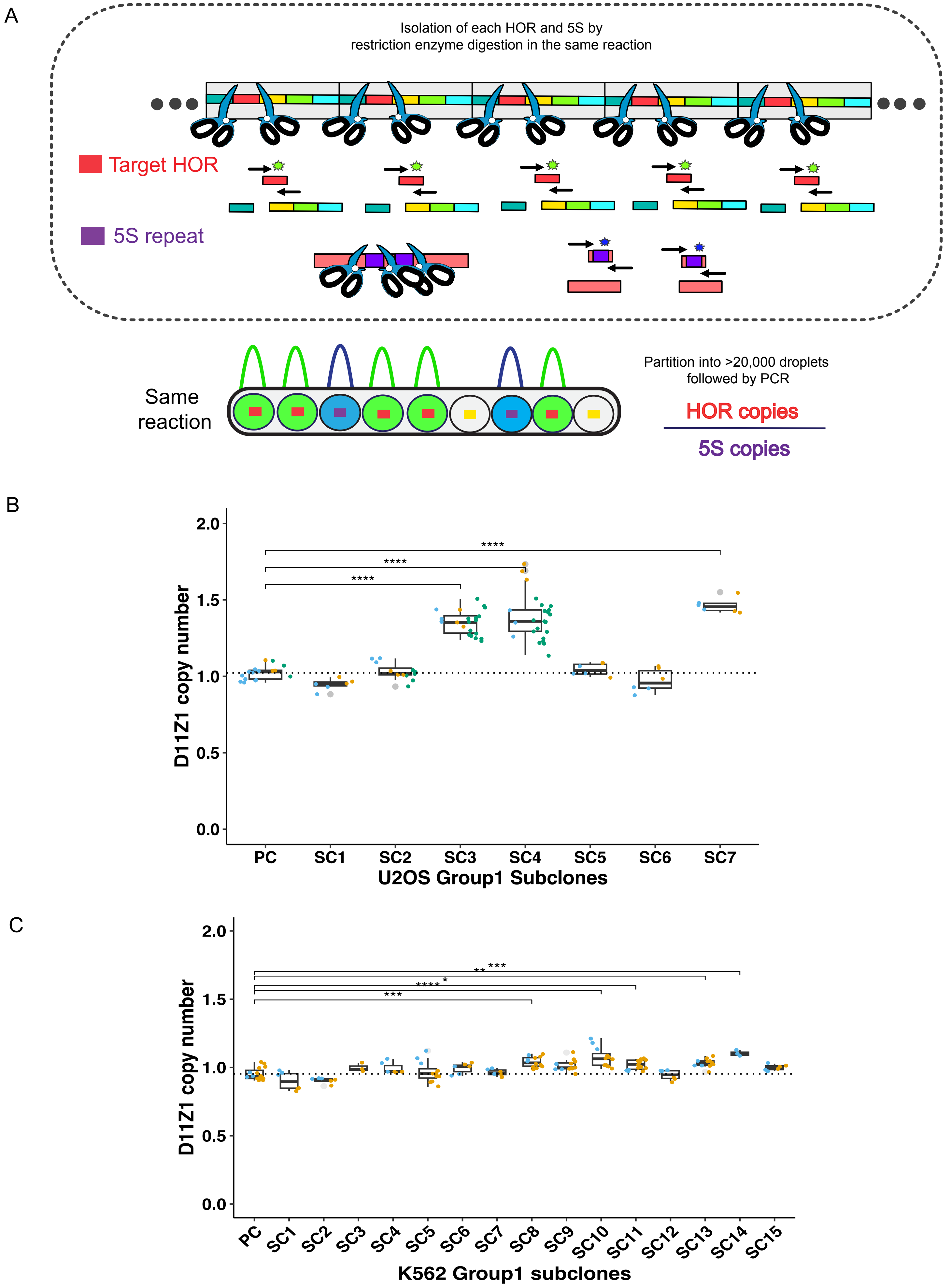

A
Isolation of each HOR and 5S by restriction enzyme digestion in the same reaction
Target HOR
5S repeat
Same reaction
Partition into >20,000 droplets followed by PCR
HOR copies
5S copies
B
D11Z1 copy number
U2OS Group1 Subclones
PC
SC1
SC2
SC3
SC4
SC5
SC6
SC7
****
C
D11Z1 copy number
K562 Group1 subclones
PC
SC1
SC2
SC3
SC4
SC5
SC6
SC7
SC8
SC9
SC10
SC11
SC12
SC13
SC14
SC15

Figure S2. D11Z1 copy numbers using 5S assay, relating to Figure 2.

(A) Schematic of 5S assay workflow. Each HOR (red square) and 5S array (purple square) from the genome that are isolated by restriction enzyme digestion are partitioned into over 20,000 droplets. Both HOR and 5S targets are simultaneously bound with the corresponding probes and amplified in the same reaction. The droplets that containing target are measured for the two different signal amplitudes and the HOR CN per 5S CN is calculated by Bio-Rad QuantaSoft with 95% confidence intervals. (B) Box-whisker plots showing the D11Z1 CN in U2OS Group1 subclones using the 5S assay. Each dot indicates a single PCR reaction, which is normalized by the mean of the parental cell (PC) HOR CN (dotted line). Colors indicate technical replicates. Asterisks indicate degree of significance in CN changes between parental cells and subclone pairs determined by Tukey's HSD test (n=8, Tukey's HSD, $P<0.05$) (C) Box-whisker plots showing the D11Z1 CN in K562 Group1 subclones using the 5S assay. (n=16, Tukey's HSD, $P<0.05$).

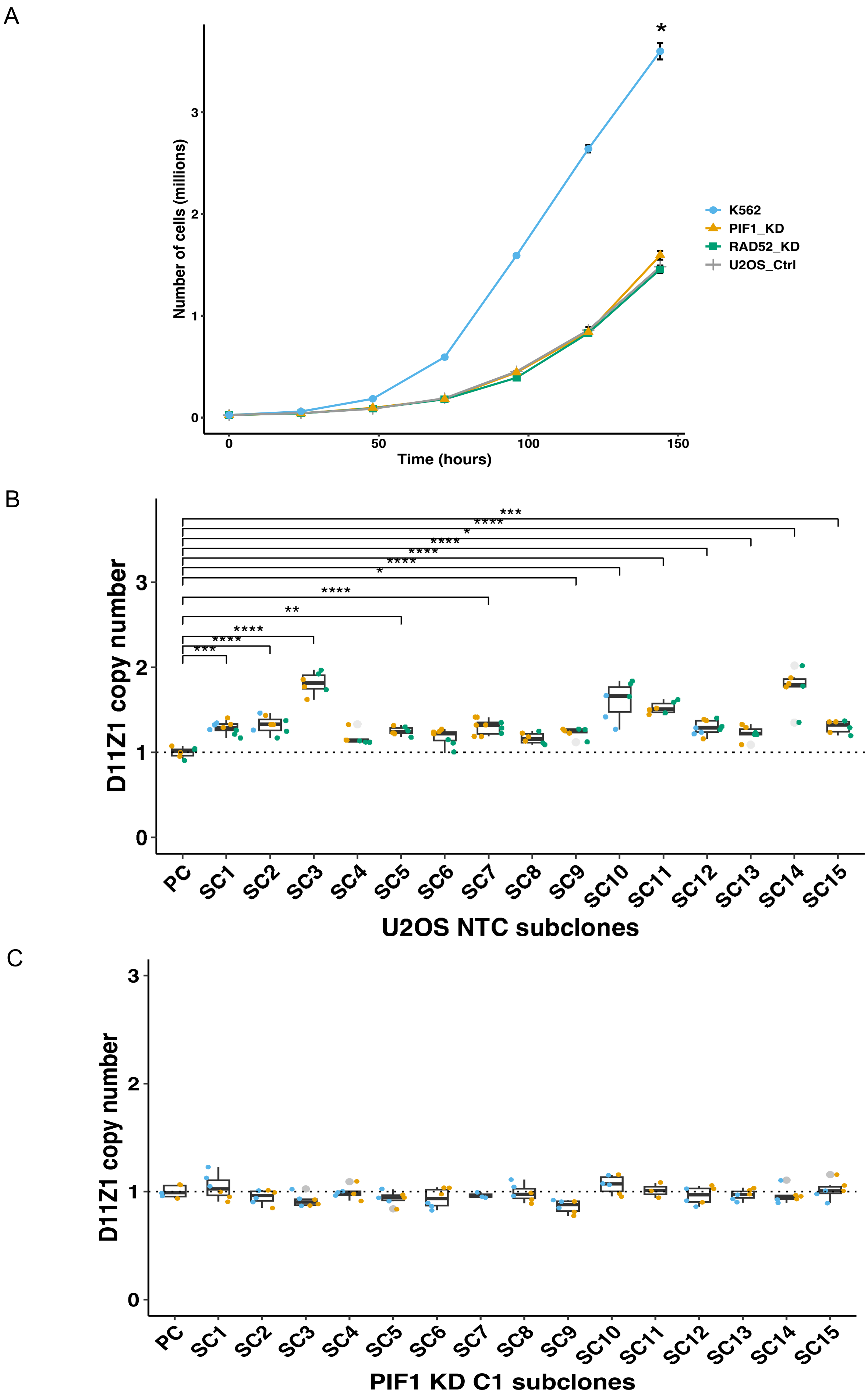

A
Number of cells (millions)
Time (hours)
K562
PIF1_KD
RAD52_KD
U2OS_Ctrl
B
D11Z1 copy number
U2OS NTC subclones
PC
SC1
SC2
SC3
SC4
SC5
SC6
SC7
SC8
SC9
SC10
SC11
SC12
SC13
SC14
SC15
C
D11Z1 copy number
PIF1 KD C1 subclones

Figure S3. No D11Z1 copy number change occurs in another PIF1 KD that has the same growth rate as U2OS control, relating to Figure 4.

(A) A line plot showing the cell numbers of U2OS control, RAD52 KD, PIF1 KD, and K562 at six time points. The colored symbols indicate the mean cell numbers of cell lines in three replicates and the bars represent ±SEM. The asterisk indicates that the K562 cell line grows differently than the other cell lines, as determined by Tukey's HSD (n=3, Tukey's HSD, $P<0.05$). (B-C) Box-whisker plot showing the D11Z1 CN in (B) NTC subclones (n=16, Tukey's HSD, $P<0.05$) and (C) $PIF1^{KD}$ C1 subclones (n=16, Tukey's HSD, $P>0.05$).
